# Bioconversion of curcumin into calebin-A by the endophytic fungus *Ovatospora brasiliensis* EPE-10 MTCC 25236 associated with *Curcuma caesia*

**DOI:** 10.1186/s13568-019-0802-9

**Published:** 2019-05-29

**Authors:** Anju Majeed, Muhammed Majeed, Nooruddin Thajuddin, Sivakumar Arumugam, Furqan Ali, Kirankumar Beede, Sebastian John Adams, Muthuraman Gnanamani

**Affiliations:** 1 Sami Labs Limited, 19/1 & 19/2, 1st Main, 2nd Phase, Peenya Industrial Area, Bangalore, Karnataka 560058 India; 2 Department of Microbiology, Bharathidasan University, Tiruchirappalli, Tamil Nadu 620024 India

**Keywords:** Calebin-A, *Curcuma caesia*, *Ovatospora brasiliensis*, Biotransformation, Baeyer–Villiger monooxygenase

## Abstract

Calebin-A is a curcuminoid compound reported to be present in *Curcuma longa* rhizome. The current study was aimed to isolate and characterize calebin-A from *Curcuma caesia* rhizome and its production through biotransformation approach using endophytic fungus. *C. caesia* rhizomes of different ages were subjected to analysis in order to investigate the age at which maximum calebin-A content is present. HP-TLC profiles, HPLC retention times and mass spectrometry detector confirmed the occurrence of calebin-A in *C. caesia* rhizomes of 12 to 14 months of age but not in rhizomes younger to 12 months. Furthermore, an endophytic fungus strain, EPE-10 that was isolated from the medicinal plant *C. caesia* was identified as *Ovatospora brasiliensis* based on morphological and molecular characteristics. This strain *O. brasiliensis* was deposited to the culture collected centre, MTCC Chandigarh, India under the Budapest treaty and was designated with the Accession Number MTCC 25236. Biotransformation process was carried out at 37 ± 0.5 °C with shaking for 7 days after addition of 0.01% w/v curcumin. Extraction of biotransformed products was done by following partition method and the extracts obtained were analyzed using HPTLC, HPLC and LCMS. The data of the study suggested that *O. brasiliensis* MTCC 25236 was found to convert curcumin to calebin-A in a time dependant manner with optimum conversion at 48 h. Furthermore, *O. brasiliensis* MTCC 25236 was found to be positive for the Baeyer–Villiger monooxygenase (BVMOs) enzyme activity which could possibly be the mechanism of this bioconversion. The results of this study for the first time indicated that the endophytic fungus identified as *O. brasiliensis* MTCC 25236 isolated from the *C. caesia* rhizome could be a possible source for naturally producing calebin-A.

## Introduction

Calebin-A is present in the *Curcuma* species along with other curcuminoids in trace amounts (Jia et al. 2017). Kim and Kim (2001) were the first to isolate and identify calebin-A from *Curcuma longa.* However, the content of calebin-A was approximately 0.001% of the total tumeric extract. Calebin-A has been shown to have the potency to prevent β-amyloid (βA) insult to neuronal cells in Alzheimer’s disease (Yan et al. 1996; Park and Kim 2002). Calebin-A also exhibited anticancer effects through the inhibition of cell signaling pathways and also down modulates the osteoclastogenesis (Tyagi et al. 2016). Furthermore, calebin-A has been reported to improve hepatic steatosis, suppress adipocyte differentiation and prevented high fat diet-induced obesity thereby suggesting the treatment of nonalcoholic fatty liver disease and obesity (Lai et al. 2015). Literature indicated that calebin-A could be a potential curcuminoid compound in managing and treating various human ailments. Due to its limited natural occurrence in *C. longa* rhizome, there is a scope to find out an alternative source of obtaining calebin-A. Of late, efforts have therefore been made to chemically synthesize calebin-A and success has been obtained (Majeed et al. 2017). However, there is still unmet need of obtaining calebin-A naturally.

*Curcuma caesia* also known as black turmeric has been used by folk healers for treating leucoderma, asthma, tumours, piles and bronchitis (Sarangthem and Haokip 2010). In India, tribal people of Adi use the rhizome decoction for curing diarrhea and Khamti tribe use the rhizome paste for snake and other insect bites (Tag et al. 2007; Kagyung et al. 2010). Black turmeric is commonly found in North-East and Central region of India and its aromatic fresh rhizomes are used to treat sprains and bruises in Indian ayurveda (Dosoky and Setzer 2018). The isolation of calebin-A from *C. caesia* has not been explored. Thus, the current study aimed to isolate and identify the calebin-A from the rhizome of *C. caesia*.

Bioconversion of chemical compounds using microbes is known to be green chemistry, which is widely used in industries for the large scale production of pharmacologically active or high value compounds. Recently, biotransformation processes have become quite promising due to the large diversity of microbes available and its wide scope in chemical reaction (Chang 2008). However, there are a few limitations associated with the biotransformation such as substrates, enzymes and stability in operation which may also be overcome through synthetic biology approach and exploration of microbial biodiversity (Bérdy 2005; Bustanussalam et al. 2015). Endophytes are the bacterial or fungal microorganisms that colonize within the plant tissues (intercellularly and/or intracellularly) without harming the host plants and reported to play a pivotal role in metabolism and biotransformation processes of certain chemical compounds involving in the biosynthetic pathways (Bacon and White 2000; Debbab et al. 2009). There are reports suggesting that the endophytes isolated from the tea plants could transform (+)-catechin and (−)-epicatechin compounds into derivative compounds 3,4 dihydroxyflavone which showed higher efficacy against cancer than the original compound, catechin (Agusta et al. 2005; Shibuya et al. 2005). To best of our knowledge, there is no literature evidence suggesting the isolation and use of endophytic microbes from *C. caesia* for the production of calebin-A, till date. Considering the importance and future prospects of the microbes as well as uses of calebin-A, it is imperative to search for endophytic microbes living in *C. caesia* rhizome. Hence, the current study focuses on the isolation of endophytic fungus from the rhizome of *C. caesia* and its use in the bioconversion of curcumin into calebin-A.

## Materials and methods

Potato dextrose agar (PDA), rose Bengal chloramphenicol agar (RBCA) and potato dextrose broth (PDB) were purchased from Himedia Laboratories Pvt. Ltd (Mumbai, India). Mercuric chloride, sodium hypochlorate, chloramphenicol, chloroform, methanol, tetrahydrofuran, ethidium bromide, hexadecyltrimethylammonium bromide (CTAB), ethylenediaminetetraacetic acid (EDTA), dimethyl sulfoxide (DMSO), β-nicotinamide adenine dinucleotide 2′-phosphate reduced tetrasodium salt hydrate (NADPH) and cyclohexanone, were procured from Sigma-Aldrich (St. Louis, MO, USA). TLC Silica Gel 60 F254 was procured from Merck KGaA (Darmstadt, Germany). Baeyer–Villiger monooxygenases (BVMOs), from recombinant *E. coli*, glycerol stock (ECS-Mo01, ECS-Mo02, ECS-Mo03, ECS-Mo04, ECS-Mo05 and ECS-Mo06) were procured from Enzymicals AG (Enzymical, Greifswald, Germany). Pure calebin-A with minimum 99% assay was obtained from the analytical department of Sami Labs Limited, Bangalore, India (Majeed et al. 2015) and used as standard throughout the study. Curcumin C3 Complex^®^ contained minimum 95% curcuminoids (~ 75% curcumin, 20% demethoxycurcumin and ~ 3.5% bisdemethoxycurcumin) was obtained from Sabinsa Corporation, 20 Lake Drive, East Windsor, NJ, USA 08520. Endophytic fungus *Ovatospora brasiliensis* strain, MTCC 25236 was isolated from *C. caesia* rhizome and deposited in the Microbial Type Culture Collection and Gene Bank (MTCC) (Chandigarh, India).

### Extraction, isolation and identification of calebin-A from the *C. caesia* rhizome

Fresh rhizomes were cleanly washed with deionizer water, sliced and dried at 50 °C in a hot air oven for 24 h. Fine powder (1.0 kg) was stirred with ethyl acetate (B.P = 77 °C), for three times of 30 min each. Final volume of 1 L of extract was added to rotary evaporator to obtain a solvent free concentrate. Solvent-free ethyl extract powder 25 g was loaded in the silica gel column chromatography and then chloroform and methanol (8:2) was used as elute solvent followed by methanol with increasing polarity. All the collected fractions were subjected to HP-TLC and HPLC for confirmation of calebin-A.

### Isolation of endophytic fungi from *C. caesia* rhizome

Healthy rhizomes of *C. caesia* were collected from the nursery of Sami Labs Limited, Bangalore, India. The plant rhizomes were analyzed and verified as *C. caesia* by the taxonomist and the voucher specimen was stored at the storage department of Sami Labs Limited with the voucher number SAMI/14/R002. The rhizomes were washed gently several times with water to remove soil and adherent particles and then dried with sterile blotting paper. Samples were sterilized by soaking into 70% alcohol for 1 min followed by sodium hypochlorate (5.3%) treatment for 5 min. Finally, the rhizomes were soaked in 0.25% of mercuric chloride (HgCl_2_) for 30 s followed by thorough rinsing with sterile distilled water to remove traces of mercuric chloride and other treatment agents and then dried using sterile blotting paper. The rhizomes were cut horizontally and vertically into small pieces using sterile blade and then carefully placed on potato dextrose agar (PDA) plate containing chloramphenicol (50 µg/ml). The plates were then incubated at 28 ± 1.0 °C for 7 to 14 days with regular monitoring for the fungus growth. The hyphal tip which grew out from the plant tissue was carefully transferred to the RBCA plates and then incubated for 7 days at 28 ± 1.0 °C. The purity of the isolated endophytic fungus was determined by the colony morphology and microscopic examination.

### Physiological profile of endophytic fungus

The pure isolated endophytic fungus strain EPE-10 was grown on RBCA and subjected to the microscopic examination (Eclipse CI, Nikon Instruments Inc, Japan). Photographic images were captured using Nikon DS Ri2 attached to a Nikon Eclipse Ci microscope. The images were processed on Nikon basic essential software. Furthermore, pure colonies were picked up and subjected to biochemical characterization based on sugar fermentation pattern in basal broth medium as per the standard method described earlier (Majeed et al. 2016).

### DNA extraction and phylogenetic analysis

Endophytic fungus strain EPE-10 was grown on PDA and the genomic DNA of the pure endophytic fungus strain EPE-10 was extracted using CTAB following the protocol of Graham et al. (1994) with slight modification. Quality of DNA was evaluated on 1.0% agarose gel and a single band of high-molecular weight DNA was observed (Fig. 7). Polymerase chain reaction was performed with primer pairs targeted to the 18S rRNA gene using the standard protocol suggested by the manufacturer and a single discrete PCR amplicon band of ~ 500 bp was observed when resolved on agarose. The PCR amplicon was purified to remove contaminants. Forward and reverse DNA sequencing reaction of PCR amplicon was carried out with NS1 (5′-GTAGTCATATGCTTGTCTC-3′) and NS4 (5′-CTTCCGTCAATTCCTTTAAG-3′) primers using BigDye™ Terminator v3.1 Cycle Sequencing Kit (Applied Biosystems, Foster City, CA) on ABI 3730XL Genetic Analyzer (Applied Biosystems). Consensus sequence of the PCR amplicon was generated from forward and reverse sequence data using aligner software (Kimura 1980). The 18S rDNA region sequence was used to carry out Basic Local Alignment Search Tool (BLAST) with the database of National Center for Biotechnology Information (NCBI) GenBank^®^ (https://blast.ncbi.nlm.nih.gov/Blast.cgi). Based on maximum identity score first ten sequences were selected and aligned using multiple alignment software program Clustal W (https://www.genome.jp/tools-bin/clustalw). Distance matrix was generated and the phylogenetic tree was constructed using MEGA 7 (Kumar et al. 2016).

### Biotransformation of curcumin into calebin-A

Freshly grown endophytic fungus strain EPE-10 was inoculated to PDB (500 ml) and incubated at 37 °C for 5 days in shaker incubator (Scigenics Biotech, Chennai, India) with 140 rpm. After incubation, Curcumin C3 Complex^®^ (50 mg dissolved in 20 ml of DMSO) was added to flask and incubated at 37 °C for 5 days in shaker incubator with 140 rpm. At 24 h interval, 100 ml of the sample was withdrawn from the flask followed by the addition of ethyl acetate (300 ml) with continuous mixing for 30 min using separating funnel (Borosil, Mumbai, India). The suspension was kept still in a stand for 30 min for separation of aqueous and organic layers. Top organic layer was carefully collected in a fresh tube and concentrated to dryness at 45 °C under vacuum using rotary evaporator (Heidolph, Schwabach, Germany). The sample was further dissolved in 20 ml of methanol. Controls respectively without adding curcumin into the medium and without adding the fungus to the medium were also used and processed similar to above. The presence of calebin-A in the extracts was identified and quantified using HPTLC, HPLC and LC–MS techniques as described below.

### High performance thin layer chromatography (HPTLC)

The preliminary identification of curcumin and calebin-A was performed by using HPTLC system (Camag, Muttens, Switzerland) comprising of Camag Linomate V semiautomatic sample applicator and Linomat syringe (100 μl). The stationary phase was TLC silica gel plates (Merck Millipore, 60 F_254_) where, 2 μl of each samples were loaded and developed using solvent system chloroform:methanol (98:2). Using scanner 3 (Camag), the plate was scanned at 280 nm with deuterium illumination. The images were captured on Camag reprostar 3 with win CATS software (ver. 1.4.3.6336) and compared the R_f_ value with the standard calebin-A.

### High performance-liquid chromatography (HPLC)

The identification and quantification of curcumin and calebin-A was performed by using Shimadzu Class Vp series HPLC system, equipped with a DAD detector (SPD-M10A Vp), binary gradient pump (LC20 AD) and, C18 column (250 × 4.6 mm, 5 µm particle size). The solvent system used for mobile phase was tetrahydrofuran: 0.6% citric acid in water (40:60) at a flow rate of 1.0 ml/min with column temperature 25 °C. The injection volume was 20 µl. Identification and quantification of the curcumin and calebin-A was done by comparing the retention time and characteristic absorption spectra from the DAD with those of the authentic standards. Data acquisition and analysis were carried out using Shimadzu LC Solution version 1.25. Samples were taken in triplicates for the analysis.

### Liquid chromatography coupled with mass spectrophotometer (LC–MS)

Analysis of curcumin and calebin-A were performed by using liquid chromatography Thermo–Finnigan surveyor coupled to electrospray ionization on a triple Quad mass spectrometer (Thermo–Finnigan LCQ Advantage Max) equipped with degasser, binary pump, auto sampler, and column heater. For analysis, 4 μl of the extract was injected. The auto sampler was cooled at 10 °C. Chromatographic separation was achieved using C18 column (250 × 4.6 mm, 5μ particle size, Thermo, BDS) with flow rate 0.3 ml/min at 25 °C. The isocratic solvent system was acetonitrile and 0.1% acetic acid in water (40:60). Electrospray ionization was performed in the negative ion mode using helium gas at a pressure of 5 psi for the nebulizer with a flow of 5 l/min and a temperature of 300 °C. The sheath gas temperature was 250 °C with a flow rate of 11 l/min. The capillary was set at 3500 V and the nozzle voltage was 500 V.

### Baeyer Villiger monooxygenases (BVMO) enzyme activity

Endophytic fungus *O. brasiliensis* MTCC 25236 was cultivated in PDB medium, containing 0.5 mg/ml of Curcumin C3 Complex^®^ (dissolved in 25 mg/ml of DMSO). The culture was incubated for 72 h at 37 ± 0.5 °C at 140 rpm. After every 24, 48 and 72 h of interval, culture was centrifuged (10,000×*g* for 10 min) to remove cells and then supernatant was collected separately. Cell free supernatant was used as a crude extracellular enzyme complex to determine the BVMO activity. In another set of experiment, the above collected cell pellet was washed three times with sterile phosphate buffer (0.1 M PBS, pH 7.5) containing 4 mM phenylmethanesulfonyl fluoride (Sigma-Aldrich). The washed cells were re-suspended in 10 ml phosphate buffer (50 mM; pH 7.5), and then were sonicated (for 45 s each cycle with 80% amplitude in cold condition) using ultrasonic homogenizer (Sartorius AG, Göttingen, Germany). The cell homogenate was centrifuged (10,000×*g*) at 4 °C and the clear supernatant was used as crude intracellular enzyme complex BVMOs activity. The in vitro BVMO activity was measured by monitoring the NADPH consumption (which is a must for BVMO activity) at 340 nm for 180 s in 1 ml cuvettes by using a UV–Vis spectrophotometer (Shimadzu Corporation, Kyoto, Japan). The assays were performed in Tris-HCl buffer (50 mM, pH 8.5) containing 0.8 mM NADPH, 10 mM cyclohexanone, by adjusting absorbance between 0.9 and 1.0 at 340 nm and followed by addition of appropriate amount of the crude enzyme extracts. One unit (U) of the enzyme activity was defined as the amount of enzyme to oxidize 1 μmol of NADPH for 1 min under the reaction conditions. Protein content was determined by following Bradford’s method, using bovine serum albumin (BSA, Sigma-Aldrich) as standard (Bradford 1976).

## Results

### Identification and quantification of naturally occurring calebin-A in *C. caesia*

**Fig. 1:**
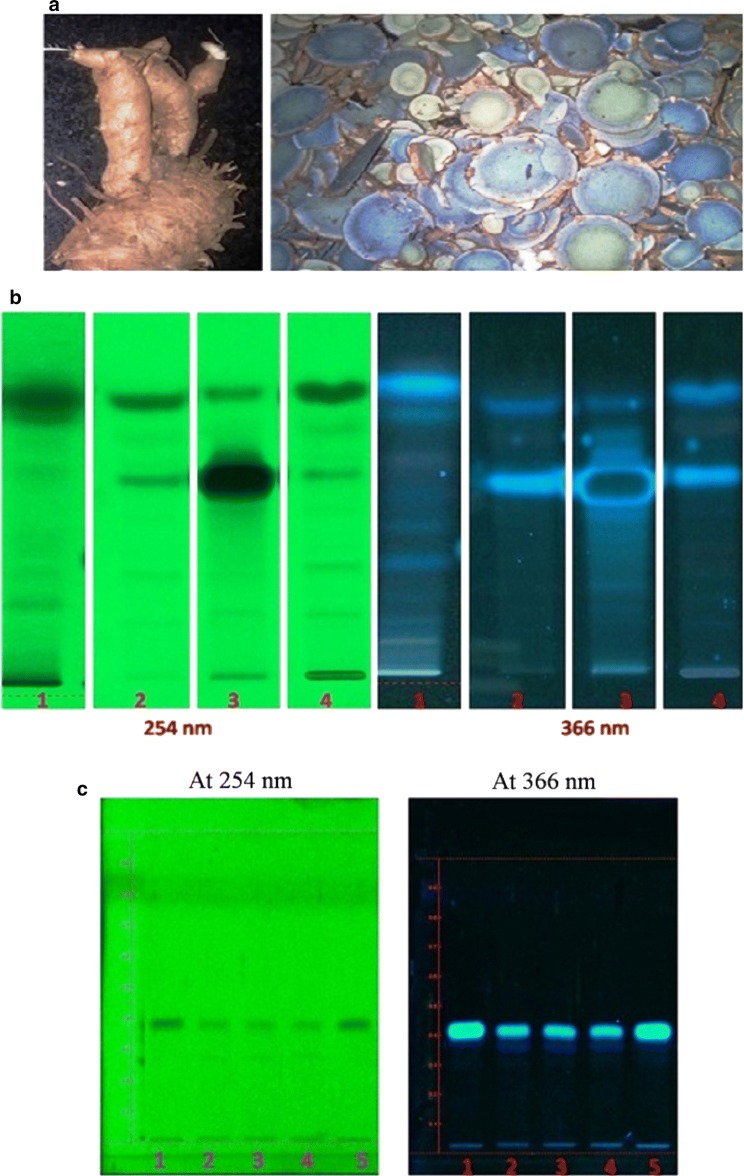
Images of **a** rhizome of *Curcuma caesia,* and chopped pieces of rhizome of *Curcuma caesia*. **b** HPTLC chromatograms. Left four lanes are viewed in 254 nm and right four lanes are viewed in 366 nm. Lanes 1 and 2 in each respectively are ethyl acetate extract and lane 4 in each methanol extract. Lanes 1 and 2 in each respectively represent extracts of 8 and 14 month old rhizomes. Lane 3 in each represents standard calebin-A. Lane 4 in each represents methanolic extract of 14 month old rhizomes. **c** HP-TLC profile of column purified calebin-A from 14 months old rhizomes of *C. caesia*. HP-TLC plates were scanned at 254 and 366 nm. Lane 1 and 5 are calebin-A standard, lanes 2 to 4 are column elutes after purification

**Fig. 2:**
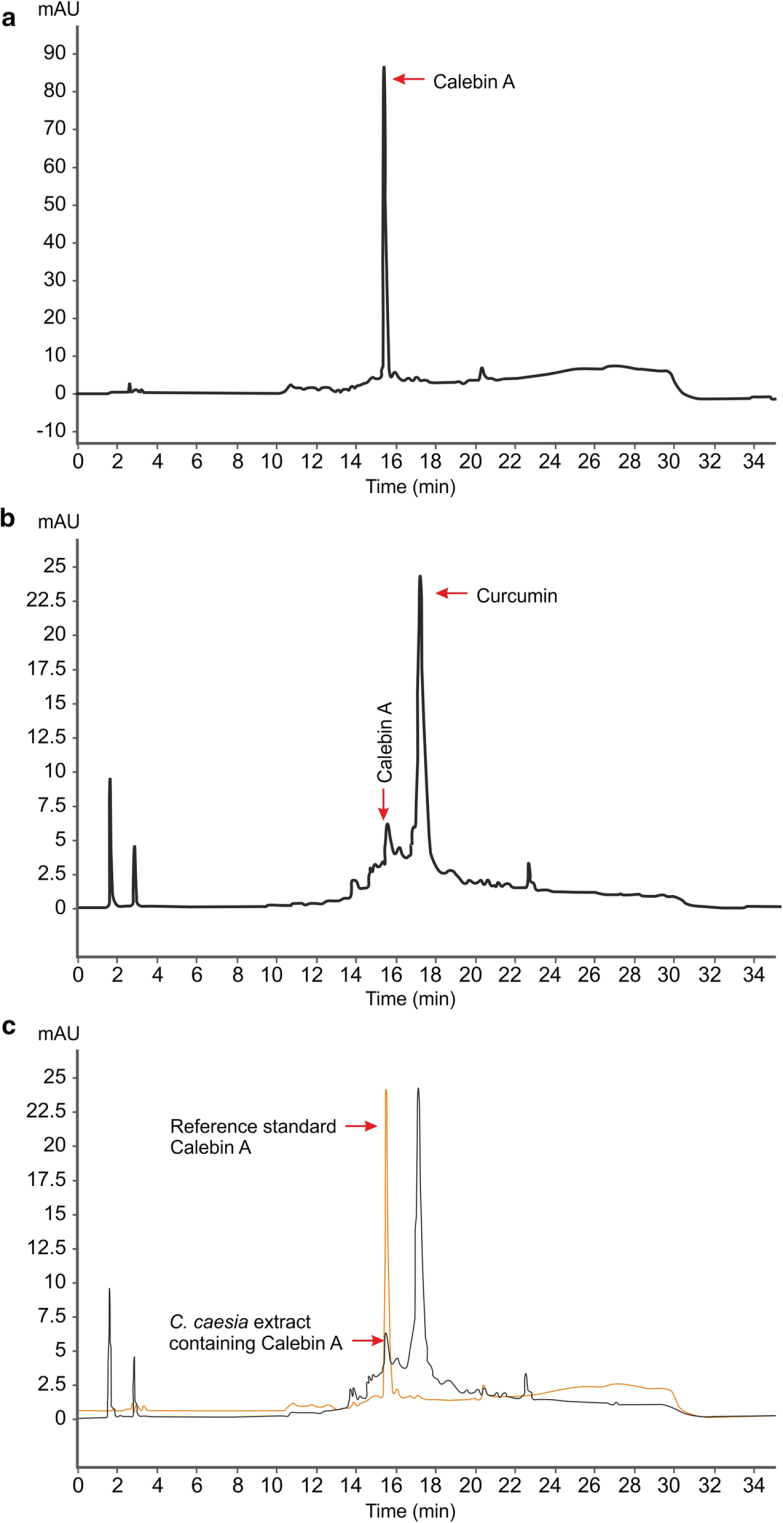
HPLC chromatograms of *C. caesia* rhizome (ethyl acetate extract) shows the presence of calebin-A. **a** Standard calebin-A, **b** ethyl acetate extract of *C. caesia* rhizome, **c** superimposed peak comparison of the standard and naturally occurring calebin-A in the rhizome of *C. caesia*

Calebin-A from *C. caesia* rhizomes (Fig. 1a) was extracted using ethyl acetate solvent and initial screening by HP-TLC/HPLC methods were followed to detect the fractions containing calebin-A in the concentrated extracts. Chromatographic separation and pooling of fractions led to samples containing enriched calebin-A. The identity and estimation of calebin-A in these extracts was proven beyond doubt by direct comparison with the standard calebin-A. HPLC retention times by UV as well as mass detector confirmed the occurrence of calebin-A in *C. caesia*. The HP-TLC analysis of the ethyl acetate and methanol extract of rhizome of *C. caesia* showed the presence of the similar bands to the standard calebin-A. The detection of the calebin-A in the extracts was observed in both 254 and 366 nm. The rhizomes of *C. caesia* at different ages were subjected to analysis in order to determine the age at which maximum calebin-A content was present. It was found from this analysis that the rhizomes of 12 to 14 months of age alone showed the presence of calebin-A and not rhizomes younger to 12 months (Fig. 1b). In order to further characterize the calebin-A band shown in Fig. 1c, column purification was done using ethyl acetate. HPLC analysis of the crude ethyl acetate extract collected from dried rhizome showed the presence of calebin-A at 15 min. The retention time matches well with standard calebin-A. The extract was further spiked with known concentration of calebin-A reference standard to quantify the compound. Based on HPLC quantification, calebin-A content was estimated to be 0.02% in the extract and 0.005% in the rhizome (Fig. 2).

### Isolation and identification of endophytic fungus from *C. caesia* rhizome

**Fig. 3:**
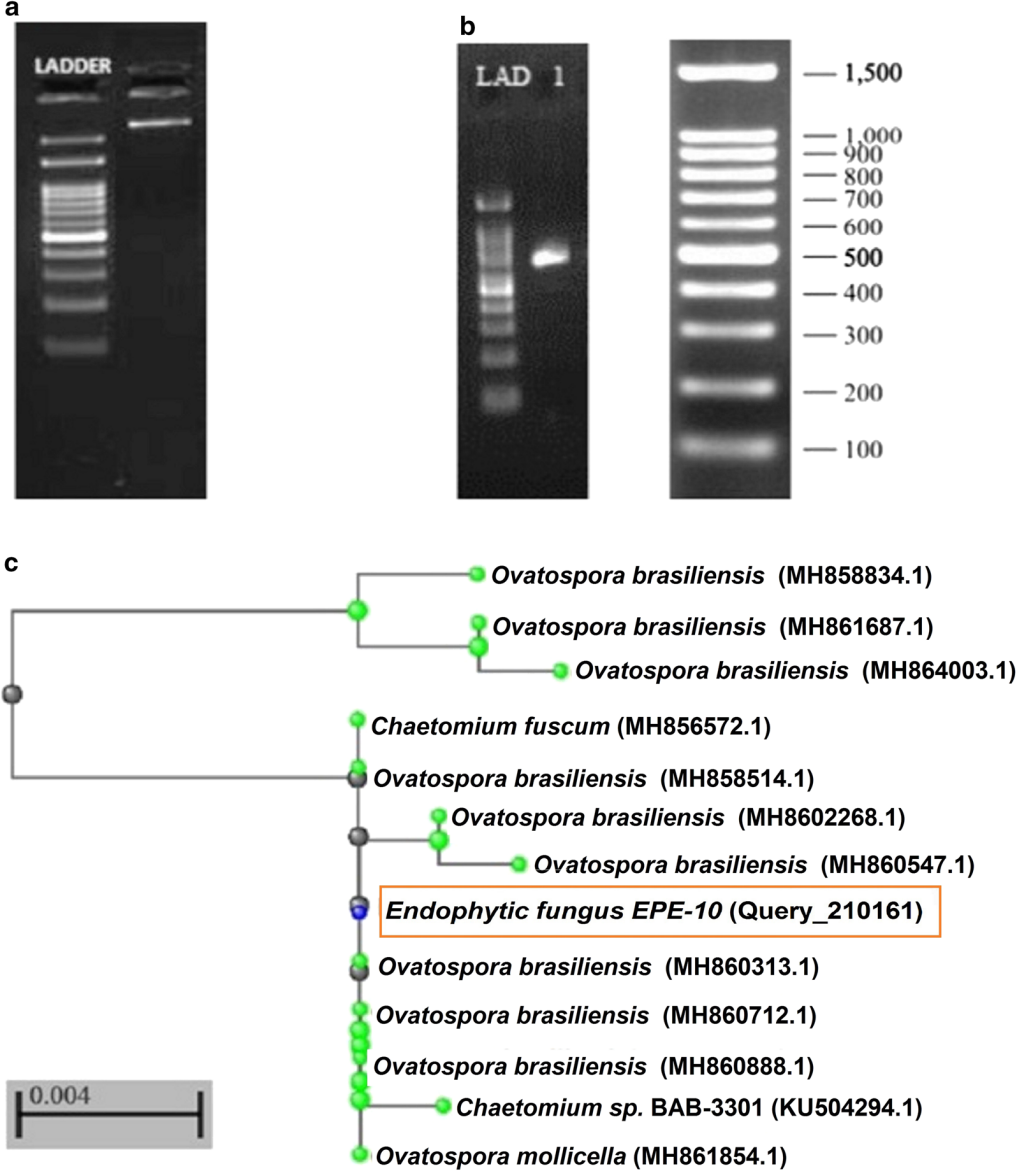
Images of gDNA extracted from *Ovatospora brasiliensis* EPE-10, MTCC 25236 (**a**) and 18S amplicon (**b**) QC data. **c** Phylogenetic tree showing the position of isolate Endophytic fungus EPE-10 with reference to related strains. All 18S rDNA sequences of related strains have been retrieved from the NCBI database; 0.004 denotes the genetic distance

**Fig. 4:**
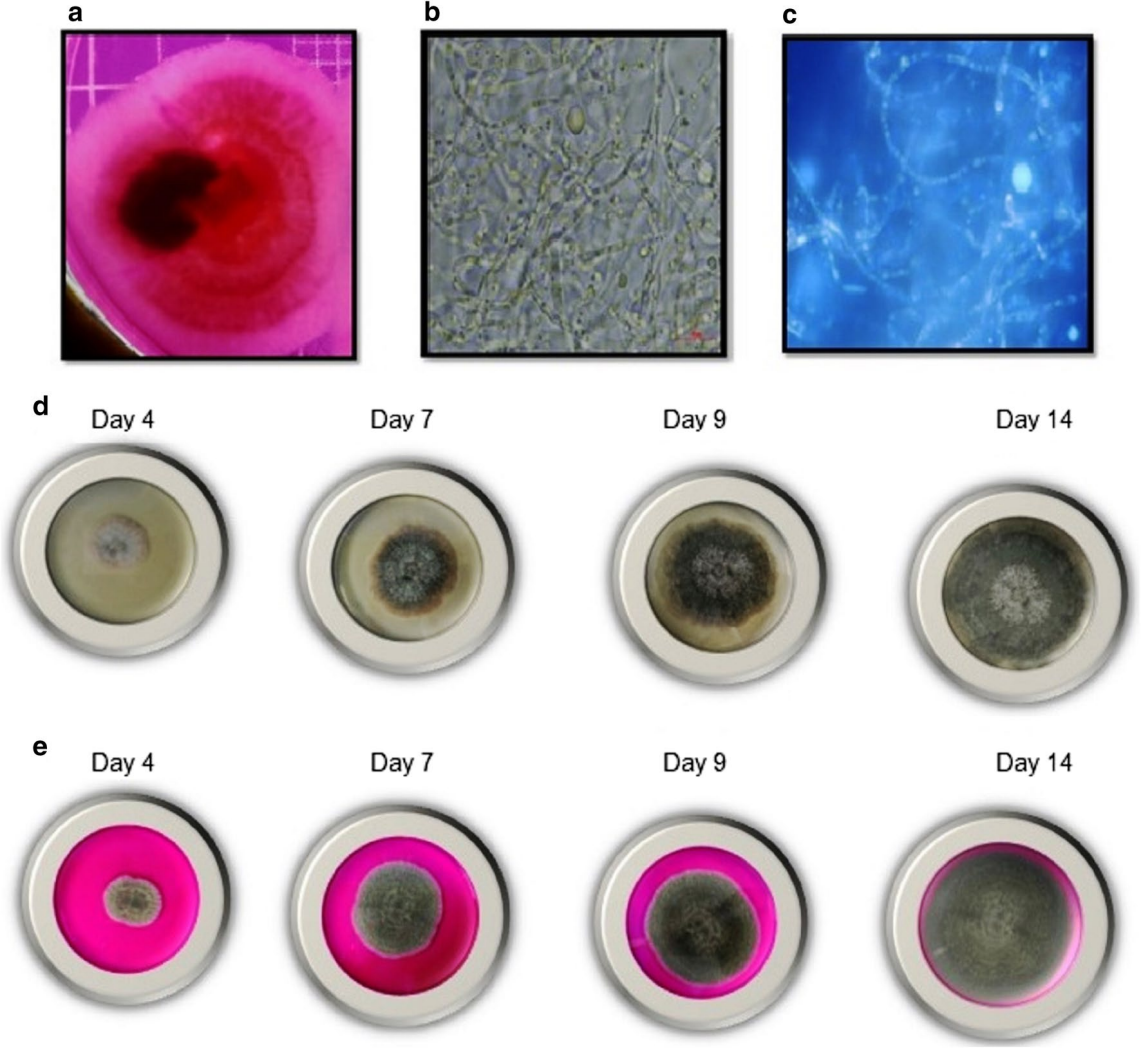
Growth of endophytic fugal strain EPE-10 **a** grew out from the explants of *C. caesia* rhizome tissue on RBCA media after 4 days of incubation at 28 °C. **b** Microscopic image of endophytic fungus *O. brasiliensis* EPE-10, MTCC 25236 using phase contrast and **c** fluorescent microscopy. **d** Images of fungus *O. brasiliensis* EPE-10, MTCC 25236 from day 4, 7, 9 and 14 days of the growth on the potato dextrose agar (PDA), and **e** rose bengal chloramphenicol agar (RBCA)

The selected 12 to 14 months old rhizomes of *C. caesia* were taken for endophytic fungus isolation, as per the procedure mentioned in the methodology. The treated explants of rhizomes were placed onto the RBCA media and the endophytic fungus EPE-10 was isolated and purified by sub-culturing (Fig. 4a). The isolated endophytic fungus was identified as *O. brasiliensis* strain EPE-10 on the basis of nucleotide sequence and morphological characteristics. The 18S rDNA sequence of strain *O. brasiliensis* EPE-10 with the length of 582 base pairs was compared against the NCBI public database, showed 100% homology with 100% query cover to that of *O. brasiliensis* EPE-10 reference strains (GenBank Accession Numbers MH858514.1) (Fig. 3a, b). The 18S rDNA sequences from the type strains and the *O. brasiliensis* strain EPE-10 were aligned and phylogenetic tree was constructed (Fig. 3c). Morphological features such as colony shape, color, and characteristics of the ascocarp, ascomal hairs, ascus and ascospores also indicated the strain EPE-10 was closely related to *O. brasiliensis* (Fig. 4b, c). The morphological characteristics of *O. brasiliensis* EPE-10 MTCC 25236 colonies were grayish white with a cottony texture, having black dots on the surface. The hyphae were septate with pale brown color. Peri-thecia was oval brown to black color and surrounded by long helical filamentous appendages. The ascospores were dark brown, oval shape with single cell with septum (Fig. 4d, e). Various biochemical characteristics and carbohydrate utilization for the growth of *O. brasiliensis* EPE-10 MTCC 25236 were summarized in Tables 1 and 2. *O. brasiliensis* EPE-10 MTCC 25236 exhibited positive tests for catalase and esculin hydrolysis. However, tested negative for the indole, MRVP and citrate test. Carbohydrate fermentation by *O. brasiliensis* EPE-10 MTCC 25236 was performed with phenol red indicator to check the acid production during fermentation. The indicator changes colour from red to yellow as pH increases during fermentation. This change in colour was recorded during the fermentation of starch, dextrose, sucrose and fructose by *O. brasiliensis* EPE-10 MTCC 25236 (Table 2). Furthermore, utilization of the carbohydrate during fermentation by *O. brasiliensis* EPE-10 MTCC 25236 was analyzed by percentage of carbohydrate initial (0 day) and after 7 days of fermentation. The utilization of starch and dextrose by *O. brasiliensis* EPE-10 MTCC 25236 after 7 days of fermentation were recorded more than 90%.

**Table 1:** Biochemical characterization of endophytic fungi endophytic fungus *O. brasiliensis* strain EPE-10, MTCC 25236

S. no.	Test	*O. brasiliensis* EPE-10, MTCC 25236
1	Catalase	Positive
2	Indole	Negative
3	Methyl red	Negative
4	Voges Proskauer	Negative
5	Citrate	Negative
6	Esculin hydrolysis	Positive

**Table 2:** Illustrates the effect of carbohydrates on the growth of endophytic fungus *O. brasiliensis* strain EPE-10, MTCC 25236

S. no.	Test	*O. brasiliensis* EPE-10, MTCC 25236	
		Growth	Color change red to yellow
1	Starch	+++	++
2	Dextrose	+++	++
3	Lactose	+++	–
4	Fructose	+++	+
5	Mannitol	+	–
6	Sucrose	+++	++
7	Maltose	+++	–

+ Mild reaction, ++ medium reaction; +++ complete reaction

### Bioconversion of curcumin into calebin-A

**Fig. 5:**
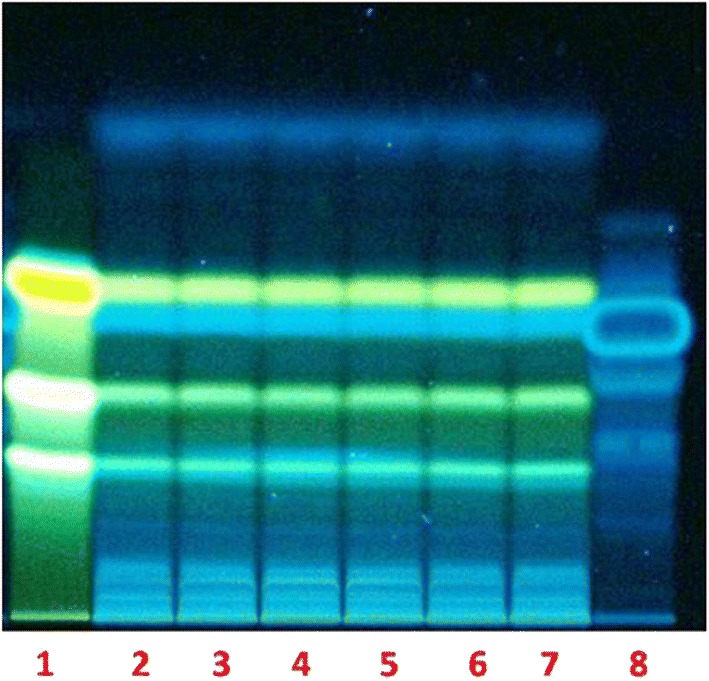
HP-TLC chromatogram of the *O. brasiliensis* EPE- 10, MTCC 25236 (ethyl acetate extract), shows the conversion of calebin-A at 366 nm. Lane 1: curcumin C3 complex; lane 2–7: ethyl aceate extract; lane 8: calebin-A standard

**Fig. 6:**
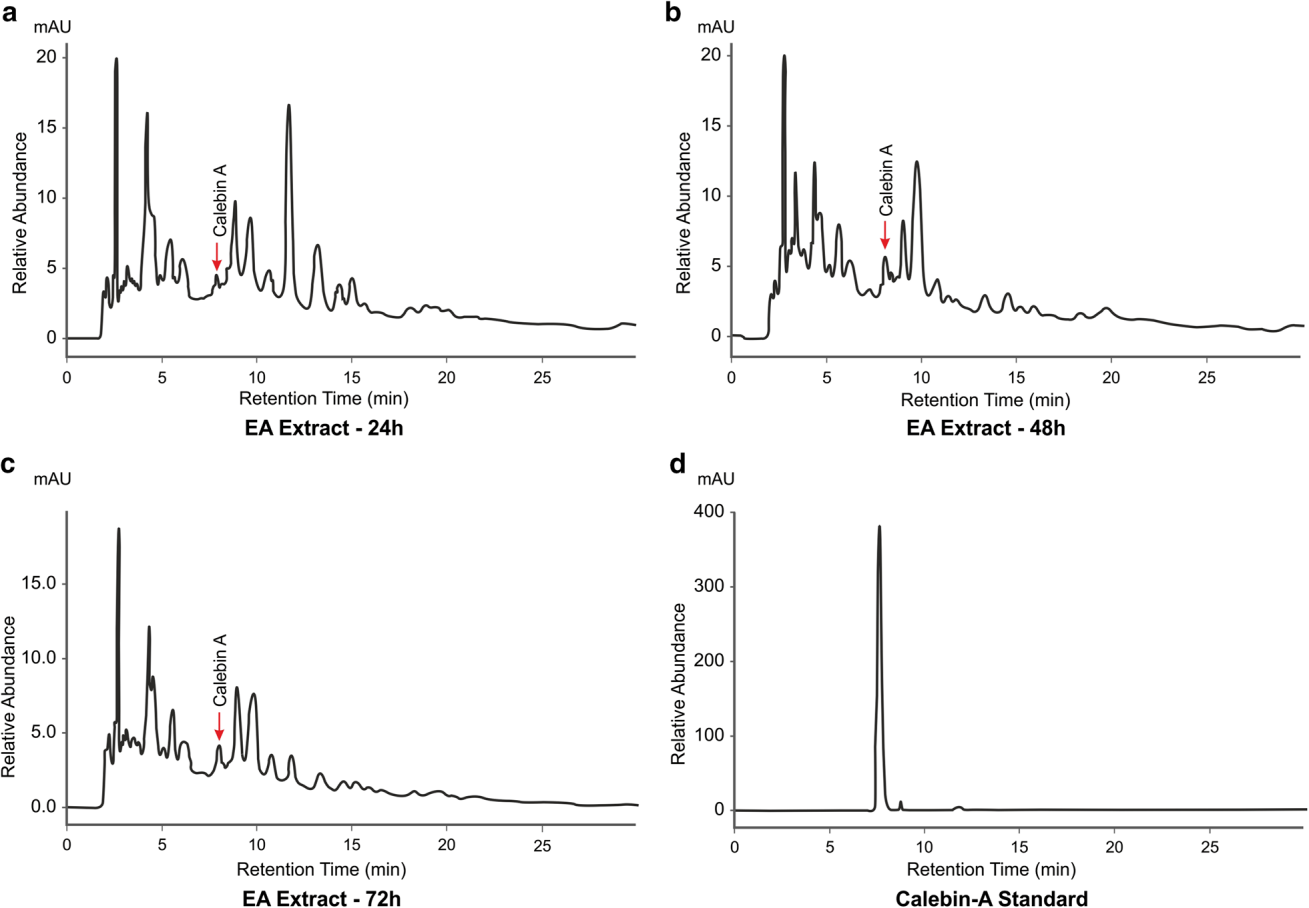
HPLC chromatogram of *O. brasiliensis* EPE-10, MTCC 25236 at different time intervals of growth and extracted with ethyl acetate **a** 24 h of growth; **b** 48 h of growth, **c** 72 h of growth, **d** calebin-A standard. The chromatogram shows the presence of calebin-A from the 24 h of growth after adding curcumin in the broth

**Fig. 7:**
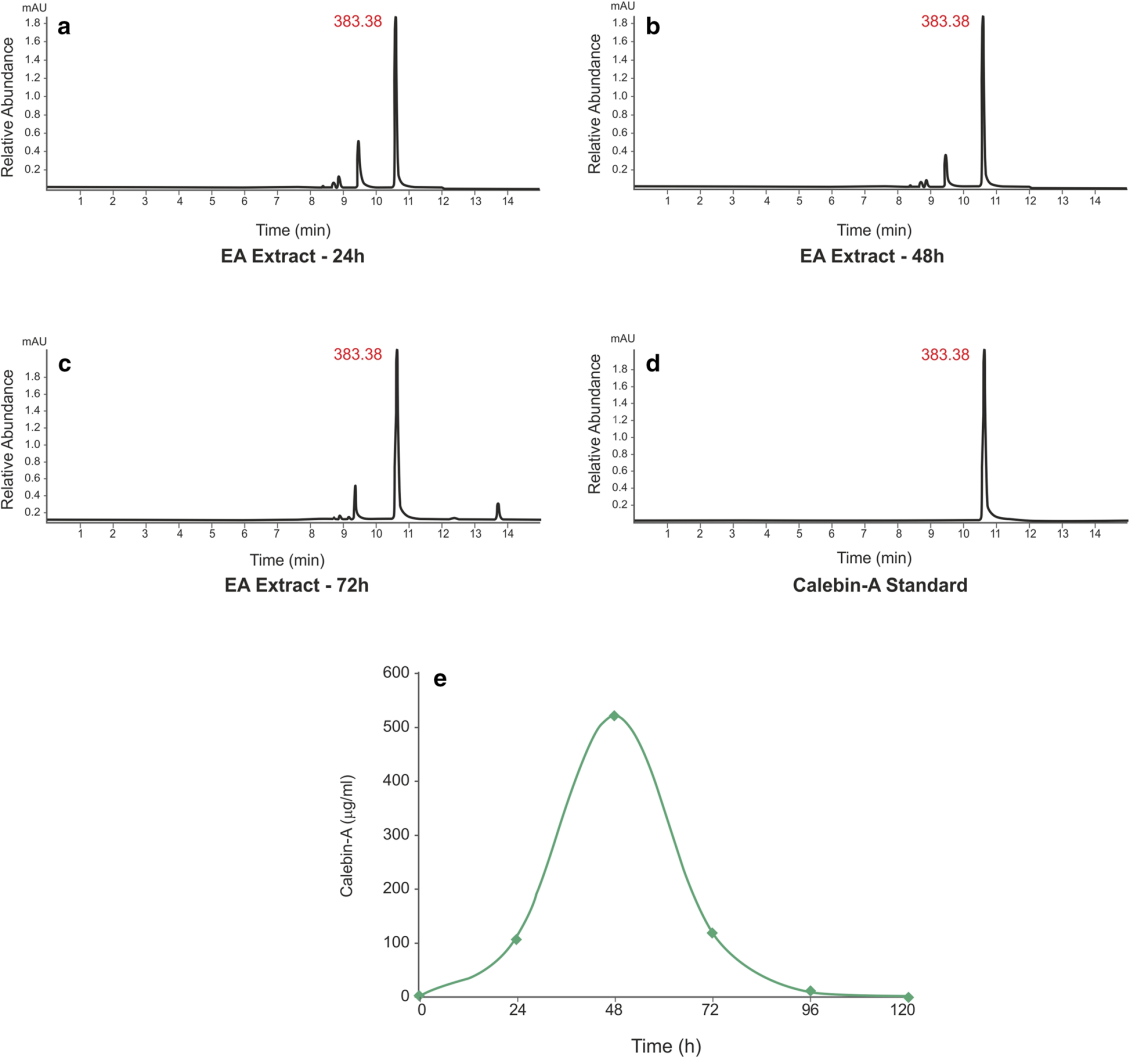
LC–MS chromatogram of calebin-A standard performed on a triple quadrupole showed similar RT 10.55 in comparison to the *O. brasiliensis* EPE- 10, MTCC 25236. The LC–MS data was more prominent in the extracts at the **a** 24, **b** 48 and **c** 72 h of curcumin added to *O. brasiliensis* EPE- 10, MTCC 25236 along with **d** calebin-A standard. **e** Conversion of curcumin to calebin-A by *O. brasiliensis* EPE- 10, MTCC 25236 at different intervals of growth. Maximum conversion was found at 48 h

The current study demonstrated the isolation of an endophytic fungal strain EPE-10 from *C. caesia* rhizomes in order to investigate the role of endophytic fungus in the bioconversion of curcumin to calebin-A. Endophytic fungus strain EPE-10 was fermented in PDB media supplemented with curcumin (0.01% w/v), extracted with ethyl acetate and then was analyzed by HPTLC, HPLC and LC–MS techniques. The HP-TLC results showed the similar band to the calebin-A standard at the same Rf. This indicated that the *O. brasiliensis* EPE-10 was able to convert curcumin into calebin-A in the suitable growth time and conditions provided (Fig. 5). The group without supplementation of curcumin in the media and *O. brasiliensis* EPE-10 did not show similar band as supplemented with curcumin, thereby, suggesting that *O. brasiliensis* EPE-10 did not produce calebin-A. The chromatogram of HPLC significantly matched with the standard of calebin-A at the RT of 7.8, the following chromatogram comparing the HPLC peak against the standard (Fig. 6). To confirm the production of calebin-A in the ethyl acetate extract, samples were analyzed using LCMS. Results of LCMS affirmed the presence of calebin-A which was higher at the 48 h of incubation suggesting that the *O. brasiliensis* strain EPE-10 had ability to produce calebin-A while using curcumin as substrate (Fig. 7a, b). Furthermore, in both the respective controls, calebin-A was not detected, further proving that the *O. brasiliensis* EPE-10 converted curcumin into calebin-A. Many endophytic fungi have been reported for their therapeutically-valued metabolites in recent era. We evaluated the extracellular and intracellular BVMOs enzyme activity of *O. brasiliensis* EPE-10 which was 12.037 U/ml and 65.986 U/ml respectively (Table 3). The enzyme which produced extracellular as well as the intracellular was comparatively same whereas, specific activity of extracellular was higher compared to intracellular enzyme. In this bioconversion study of curcumin to calebin-A in the presence of *O. brasiliensis*, BVMO enzyme activity was analyzed by using cyclohexanone as a substrate and NADPH was used as the cofactor.

**Table 3:** Specific activity of BVMO enzyme at different time intervals of curcumin to calebin-A bioconversion by *O. brasiliensis* EPE-10, MTCC 25236 intracellular and extracellular enzyme

S. no.	Time (h)	Activity (U/ml)		Protein content (mg/ml)		Specific activity (U/mg)	
		Intracellular	Extracellular	Intracellular	Extracellular	Intracellular	Extracellular
1	0	0.0	0.0	0.0	0.0	0.0	0.0
2	24	0.723	0.780	0.028	0.128	26.308	6.116
3	48	1.897	1.407	0.029	0.117	65.986	12.037
4	72	0.619	0.732	0.041	0.172	15.236	4.256

## Discussion

Calebin-A, a pharmacologically important compound found in the *C. longa*, showed higher ability to inhibit the proliferation of HepG2 cells than its structurally similar curcuminoids i.e. curcumin (Chen et al. 2009). Furthermore, it also showed higher protection of PC-12 and other susceptible cells from the β-amyloid insult compared to curcumin (Majeed et al. 2015). Literature also suggested that it could also be a potent compound for the management of human gastric and other MDR cancers (Li et al. 2008). The natural occurrence of the calebin-A has been reported by the various researchers from the *C. longa* rhizome. However, the quantity reported in *C. longa* rhizome is very minimal (0.001% w/w). To best of our knowledge, there is no report suggesting the presence of calebin-A from the *C. caesia* rhizome. Thus, the current study investigated the presence of the calebin-A in the *C. caesia* rhizomes. Data of our study revealed that calebin-A content was estimated to be 0.05% (w/w) in the extract and 0.005% (w/w) in the rhizome of *C. caesia.* This is the first time we report the presence of calebin-A in the chemical constituent of *C. caesia* and the content of calebin-A was 5 times higher than the earlier reported by Kim and Kim (2001) in *C. longa* rhizome. Moreover, Kim and Kim (2001) were the first to isolate and identify calebin-A from species *C. longa.* The study also suggested that the presence of calebin-A in the *C. caesia* was age dependant. It is likely that the age of the rhizome is the crucial factor in calebin-A production. The reason for calebin-A production in older rhizome is not yet known and may require further studies. However, it is likely that aging may induce the biosynthesis of calebin-A. Thus, this suggested that occurrence of the calebin-A is age dependant and may require very specific age to obtain calebin-A from *C. caesia.*

Endophytic microbes that live in the medicinal plant tissue without harming its host may produce bioactive compounds or other useful metabolites due to co-evolution or genetic transfer from the host plant to endophytic microbes (Khare et al. 2018). Thus, the current study demonstrated the isolation of endophytic fungus from the rhizome of medicinal plant *C. caesia* and was identified as *O. brasiliensis* strain EPE-10 on the basis of nucleotide sequence and morphological characteristics. Previously, several endophytic fungi have been isolated from the medicinally important plants such as *Maytenus hookeri* (Ni et al. 2008), *Ginkgo biloba* (Qin et al. 2009), rhizome of *C. wenyujin* (Wang et al. 2012), *Withania somnifera* (Kumar et al. 2013), *Houttuynia cordata* (Pan et al. 2016). However, to best of our knowledge, there is no report in the literature suggesting the isolation of endophytic fungus *O. brasiliensis* from the rhizome of *C. caesia.* Hence, the current study is the first report on the isolation and identification of endophytic fungus *O. brasiliensis* from the rhizome of *C. caesia*.

The production of bioactive and pharmacologically active or high value compounds through bioconversion/biotransformation processes is known to be green chemistry and has great industrial interest due to its large scale production feasibility (Bérdy 2005; Bustanussalam et al. 2015). Endophytic microbes are known to play a pivotal in the biosynthesis of some medicinally important compounds (Ding et al. 2006; Wijeratne et al. 2006; Lösgen et al. 2007; Khumkomkhet et al. 2009). Thus, the current study investigated the role of endophytic fungus *O. brasiliensis* from the rhizome of *C. caesia* in the production of calebin-A using curcumin as a substrate. The bioconversion of curcumin to calebin-A was confirmed by various techniques (HP-TLC, HPLC and LCMS). HPLC and LCMS chromatogram also revealed that the *O. brasiliensis* strain EPE-10 was able to convert the curcumin into calebin-A with the maximum conversion at 48 h of incubation. Curcumin is also a curcuminoids which is found majorly in the rhizome of *C. longa* and has similar chemical structure as calebin-A. Furthermore, the therapeutic properties and simple structure of curcumin makes it an ideal target for biotransformation to yield biologically important products i.e. calebin-A. Microbial conversion of ‘natural’ curcumin could also be valuable resource and reference for medical and food sciences as reported earlier (Choudhary et al. 2011). The findings of this study suggested that the biotransformation route for obtaining natural calebin-A may hold industrial potential while using curcumin as the substrate. However, the results of this study are indicative and require further optimization and scale-up studies in order to establish commercially economical process to obtain natural calebin-A. Nevertheless, this is the first report to demonstrate the production of natural calebin-A through microbial biotransformation.

Baeyer–Villiger monooxygenases (BVMOs, EC 1.14.13.X) are the flavoenzymes which catalyze the electrophilic oxygenation of various heteroatoms and also insertion of an oxygen atom between a C–C bond in ketones and aldehydes which is very similar to the chemical Baeyer–Villiger oxidation (Zhang et al. 2018). BVMOs are known to play a pivotal role in the degradation of ketones, steroid and metabolism of terpenoids. It may also to be noted that BVMOs are considered as one of the most essential biocatalysts for organic synthesis. Thus, we speculated that *O. brasiliensis* EPE-10 may also possess BVMOs enzyme activity which could be responsible for the conversion of curcumin to calebin-A. Results suggested that the BVMOs enzyme was produced by the *O. brasiliensis* which may effectively catalyze the conversion reaction of curcumin into calebin-A. The experimental data reveals that the *O. brasiliensis* was able to produce the curcuminoid compound calebin-A by converting curcumin in the presence of BVMOs enzyme. However, further studies may be needed to elucidate the mechanism of action and role of BVMOs enzyme in the conversion of curcumin to calebin-A.

In conclusion, this study demonstrated for the first time the presence of the calebin-A in 12 to 14 months aged rhizome of *C. caesia*. Furthermore, endophytic fungus isolated from the *C. caesia* rhizome, was identified as *O. brasiliensis* strain, EPE-10 MTCC 25236 based on morphological characteristics and 18S rDNA sequence analysis, also have the potency in converting the calebin-A using curcumin as the substrate. Although further scale-up studies are needed in order to develop a commercially economical process, this experiment laid a light path for the production of calebin-A at the large scale since, it is available in minor quantity in *Curcuma* species.

## Data Availability

The datasets supporting the conclusions of this article are included within the article.
